# Supplementary material for: Are carbon emissions trading and green financial instruments synergistic? -Comprehensive quantitative research based on content analysis
Source: PLoS One. 2024 Mar 7;19(3):e0298601. doi: 10.1371/journal.pone.0298601 (PMC10919601; doi:10.1371/journal.pone.0298601)
Supplement: S1 Data — (ZIP) [file pone.0298601.s001.zip › Data/地级市Z_F工作报告.zip!/╝¬┴╓/░╫╔╜╩╨/└┤╫╘╣1⁄2╓┌║┼í╛┬φ┐╦╩2╛▌═°í┐_2005.docx]

各位代表：

　　白山市第四届人民政府任期已满。我受市人民政府委托，向第五届人民代表大会第一次会议报告工作，请予审议。并请各位政协委员提出意见和建议。

　　一、过去五年工作的回顾

　　2000年以来，我们在省委、省政府和市委的正确领导下，在市人大、市政协的监督和支持下，团结带领全市各族人民，迎难而上，真抓实干，实现了国民经济平稳增长，社会各项事业较快发展，人民生活水平进一步提高，基本完成了白山市第四届人民代表大会确定的各项任务。

　　五年来，全市生产总值年均增长9.9%，其中一、二、三产业增加值分别增长6.2%、11.26%、10.3%。一般预算全口径财政收入年均增长6.1%。累计完成全社会固定资产投资177.8亿元，年均增长11.7%。社会消费品零售总额年均增长7.6%。全市有455户国有和集体企业完成了不同形式的改革。民营经济总户数达到4.19万户，其中规模以上企业71户，从业人员14.7万人。新批外商投资企业35户，总投资7908万美元，实际利用外资2643万美元，引进国内资金19亿元，完成外贸进出口总额3.28亿美元。全市新开发就业岗位9.4万个，增加城镇就业7.5万人，城镇登记失业率控制在4.2%以内。累计发放社会保障、社会救助、社会救济资金25.8亿元，保障能力进一步增强。城镇居民人均可支配收入、农民人均纯收入年均分别增长11.5%和7.4%。

　　五年来，基础设施建设成效显著，社会各项事业长足发展。完成投资40.8亿元，新增公路里程1915公里，北线一级、长白山西坡公路等重点项目竣工，83%的乡镇和55%的行政村通了油（水泥）路。投入资金34.8亿元，完成了市中心广场、金河花园小区、长白山大街、卫国路公铁立交桥等一批重点城市建设项目。曲家营水利枢纽、浑江干流堤防、鸭绿江界河堤防等一批重点水利水电项目进展顺利，完成了农村电网改造二期工程。国民经济信息化步伐加快，覆盖全市的现代通信网络基本形成。深入实施了科教兴市战略，一批推动经济发展的科技项目列入国家和省科技发展计划；投入教育经费19.24亿元，比前五年增长53.29%。广播电视覆盖率和播出质量进一步提高。图书馆、文化馆、滑雪场、中心血站、传染病医院、疾控中心等一批文化、体育、卫生基础设施相继建成。公共卫生工作全面加强，完善了突发公共卫生事件应急预案，有效防止了非典和禽流感疫情的传入。计划生育政策得到全面落实，人口自然增长率控制在3.45‰。妇女儿童和关心下一代工作得到进一步加强。切实维护了民族团结。依法加强了对宗教事务的管理。

　　五年来，围绕转变政府职能，顺利完成了政府机构改革。适时进行了行政审批、会计核算、土地收储三项制度改革，全面推行了政务公开，强化了政府的服务职能，进一步优化了经济发展软环境。不断完善社会治安"打防控"体系建设，严厉打击"法轮功"等邪教组织非法活动，认真处理群众来信来访，狠抓以煤矿为重点的安全生产，保持了政治安定和社会稳定。

　　五年来，统计、审计、物价、环保、水文、气象、人防、民兵预备役、地震、外事、地方志、档案、残疾人等工作也为全市经济社会发展做出了应有的贡献。

　　2004年，我们把加快发展作为执政兴市的第一要务，以科学发展观统领资源型城市发展接续产业示范基地的各项工作，坚持工业立市、工业强市，积极推进经济增长方式的转变，实现了快速增长。全市完成生产总值128.6亿元，比上年增长14.7%，创历史最高增幅，人均GDP首次超过1000美元，达到1183美元。规模以上工业完成增加值39. 6亿元，增长29%，实现净利润4.27亿元，增长1.37倍；旅游社会总收入6.14亿元，增长1.5倍；全社会固定资产投资完成45.1亿元，增长29%，增幅均创历史最高水平。全口径财政收入实现9.13亿元，增长20.3%，增幅创1997年以来新高，继抚松县之后，又有江源县、临江市和八道江区财政收入突破亿元。全市社会消费品零售总额完成49.7亿元，增长8.9%。完成外贸进出口总额8947万美元，增长31.4%。

　　--抢抓机遇，谋划和建设一批左右全市经济社会发展形势的大项目。切实把项目建设作为政府抓经济工作的核心，对市属重点项目都组建一套班子、明确一个目标、拨付一笔经费、完善一套奖惩机制，保证了项目建设的成功率。各级政府都落实了项目建设责任制，主要领导抓最大、最难的项目，全市项目建设出现了前所未有的好形势。全年投资千万元以上续建和新开工项目78项，完成投资25亿多元。农夫果园饮料系列产品深加工、5000吨碳化硅微粉、临江聚宝电站等14个项目竣工投产。60万吨焦化工程正在抓紧建设。大横路钴矿已进入冶炼试验阶段。浑江发电公司60万千瓦机组扩建一期工程，已落实了投资主体，完成了主设备招标。60万吨林浆纸一体化项目，正在做开工前的准备工作。奥迪C6轿车门内板焊接总成、超微细二氧化硅等6个项目，被国家发改委批准列入第二批老工业基地调整改造国债项目。公路建设完成投资13.6亿元，在建里程1180公里，投资额和在建规模超过以往任何一年。南线一级公路、老岭隧道及连接线、鸭绿江边防公路、长白山南坡公路等9个重点工程进展顺利。辉南至靖宇地方铁路已部分铺轨。年内又有4个乡镇、64个行政村通了油（水泥）路。长白山机场开工前的准备工作已接近尾声。营城子至松江河高速公路、环长白山公路等一批重点项目的前期工作有突破性进展。

　　--围绕体制和机制创新，积极稳妥地推进了各项改革。在市直部门抽调46名业务骨干，组成16个工作组，深入企业具体指导，全市有94户企业完成了"一个置换、两个转变"、净资产量化、产权出售、股权转让等不同形式的改革。通过政策性破产、诉讼清偿、核销呆帐和打包回购债权，为589户企业核销银行本息20.9亿元。通化矿务局湾沟、苇塘两矿破产即将终结，完成了社会职能向地方政府移交工作。农村税费改革进一步深化，通过免征农业税、取消农业特产税和实施粮食直补、良种补贴等政策性措施，切实减轻了农民负担，增加了农民收入。继续推进医疗保险制度改革，全面启动了工伤保险。完成了市区两级公安机关警务改革，一线警力增加80.8%。

　　--着眼于引进战略投资者，招商引资取得明显成效。加强对"招商引资年"活动的组织领导，积极走出去、请进来，认真组织了赴北京、深圳和韩国等地的招商引资活动，积极参加省组织的各项大型经贸活动。以我市被国际饮水资源保护组织命名为"中国·白山国际矿泉城"为契机，成功举办了首届中国·白山国际矿泉节和天然矿泉水国际论坛，吸引了30个国家和地区的国内外来宾480多位，集中展示了我市的投资环境和资源优势。成功引进中国电力投资集团公司、中国包装总公司、上海中产经投资有限公司等战略投资者，与我市在资源深度开发、基础设施建设等方面进行全方位、多层次的合作。全年共签订合资、合作项目127项，意向总投资85亿元，招商引资实际到位资金10.26亿元。

　　--加大建设和整治力度，发展环境进一步优化。重新修编了《白山市城市总体规划》，启动了江北新区开发工程，星泰大桥、人行过江悬索桥及拦江蓄水钢板闸、物流中心、大通沟供水改造等一批城市重点项目进展顺利，基本完成了卫国路、南平街、通沟街与南线接口的管线铺设，采煤沉陷区综合治理工程已完成开工准备。新建和改造50条街路，为26条巷道和近百栋居民楼安装了"两门两灯"。以建设诚信政府和服务型政府为重点，继续深化行政审批制度改革，进一步推行政务公开，充分发挥了市政务服务中心的作用，各县（市）区政务服务中心也在抓紧建设。市长公开电话成为反映社情民意的"绿色通道"。全市建设工程招投标、经营性土地挂牌拍卖、采矿权出让、产权交易和政府采购等方面工作得到进一步规范。在市县两级重点行业和部门开展了民主评议政（行）风和软环境"双评"活动。加大了对涉软案件的查处力度，全市共受理举报投诉141件，其中立案查办33件，追究法律和纪律责任 25人。

　　--精神文明建设不断加强，社会各项事业繁荣发展。深入实施人才兴业战略，积极开展"解放思想，二次创业"活动，大力弘扬长白山文化，激发了全民创业热情。成功举办了第五届全市运动会。结合加强未成年人思想道德建设，对网吧进行了专项整治。通过开展文明清洁城市百日综合整治等一系列精神文明创建活动，营造了健康文明、奋发向上的社会氛围。教育改革进一步深化，教学质量稳步提高。完成了市传染病医院二期工程建设。加大了食品、药品监管和检查力度。人口自然增长率控制在2.6‰。强化了环境保护和污染治理。社区基础设施建设和服务功能得到加强。

　　--高度重视群众切身利益，人民生活质量不断提高。认真实施就业和再就业、社会保障、扶贫解困三项工程，努力解决涉及群众切身利益的突出问题。制定了全民创业促就业优惠政策，进一步完善就业服务体系。全市新开发就业岗位3.4万个，新增就业3万人，当年劳务输出2.4万人，城镇登记失业率控制在4%以内。争取国家配套补助资金额度1.08亿元，启动了完善城镇社会保障体系试点工作，128户企业进入试点程序。全市共发放养老保险金、失业保险金、最低生活保障金6.1亿元，发放救灾和救济金4920万元。城乡居民收入不断增加，城镇居民人均可支配收入7265元，农民人均纯收入3000元，分别增长10.8%和17.4%。

　　--强化社会治安、安全生产和信访工作，保持了政治安定和社会稳定。始终保持对各类刑事犯罪的高压严打态势，全市刑事案件发案率比上年下降6.7%。连续开展了两个"百日安全整治"活动，全市各类安全事故比上年下降17.6%。实现连续25年无重大森林火灾。进一步加强和规范信访工作，认真落实领导接待日和律师参与信访工作等制度，变上访为下访，努力解决群众反映的实际问题。

　　--进一步提高了破解难题和应对复杂局面的能力，政府自身建设得到加强。深入实际调查研究，着力解决大宗地工产品运输困难、高耗能企业电价、困难企业参加社会保障试点等一系列难题。多渠道向上争取资金15.9亿元，比上年增长146.4%，有效缓解了经济和社会发展的资金困难。制定了《白山市人民政府突发公共事件总体应急预案》，规范了突发性自然灾害、公共事件、社会安全事件的预防和处置办法。面对鸭绿江流域出现的特大洪水，及时组织抗洪抢险，妥善安置受灾群众，没有发生一起人员伤亡事故。认真贯彻实施《行政许可法》，重新修订《政府工作规则》，制定和完善了各项配套制度。建立了跟踪督查与重点督查相结合的工作机制，确保各项任务落到实处。全面落实党风廉政建设责任制，加大了违法违纪案件的查处力度，廉政建设不断加强。强化了普法和依法治市工作。市政府自觉接受人大、政协的法律监督、工作监督和民主监督，认真及时办结人大代表议案、建议和政协委员提案，办理质量明显提高。

　　各位代表，过去五年所取得的成就，凝聚了全市人民的智慧和汗水。在此，我代表市政府向全市各族人民，向市人大、市政协、各民主党派、人民团体及社会各界人士，向中省属企事业单位的广大干部职工、人民解放军指战员和武警部队官兵，表示衷心的感谢和崇高的敬意！

　　在肯定成绩的同时，我们也清醒地认识到，当前我市经济和社会发展仍存在一些突出的矛盾和困难，政府工作还存在一些问题和不足。主要表现在：经济总量小，产业链条短，资源转化层次低，民营经济发展缓慢，产业结构、产品结构、所有制结构调整的任务非常艰巨；投资增长与建设资源型城市发展接续产业示范基地的要求不相适应；随着工资、社会保障等刚性支出不断增加，财政收支矛盾十分突出。由于林、煤等资源开采型企业的结构调整，下岗失业人员逐年增多，就业、再就业和社会保障面临巨大压力；干部群众收入和消费水平较低，低收入群体比重较大，部分群众的生活还很困难。全社会的文明诚信程度不高，创业氛围不浓，发展环境亟待改善。部分政府工作人员精神状态不佳、工作作风不实、综合素质不高，为经济发展服务的意识和破解难题的能力有待进一步提高；廉政建设还需继续加强。对上述困难和问题，我们一定高度重视，采取有效措施，认真加以解决。

　　二、今后五年的主要任务

　　今后五年是我市加快推进全面建设小康社会进程、实现资源型城市发展接续产业示范基地目标的关键时期。当前，全球经济一体化进程不断加快，国家振兴东北老工业基地各项措施深入实施，新一轮国内外生产要素优化重组、产业转移，为我们进一步加快发展提供了新的契机。我们必须紧紧抓住并切实用好本世纪头20年的重要战略机遇期，以超前的思维和高昂的精神状态，锐意进取，勇于创新，在应对挑战中把握主动，在扬长避短中争创优势，在超越自我中寻求突破，以新的作为推进白山经济社会的快速发展。

　　今后五年政府工作的总体要求是：以邓小平理论和"三个代表"重要思想为指导，牢固树立和全面落实科学发展观，紧紧抓住振兴东北老工业基地的历史机遇，突出工业立市，坚持把加快发展、强市富民作为政府全部工作的主题，围绕资源型城市发展接续产业示范基地的奋斗目标，加快改革开放进程，加大投资拉动力度，加速结构调整步伐，积极推进经济增长方式转变，不断增强综合经济实力，努力繁荣各项社会事业，进一步提高人民生活水平，着力构建社会主义和谐社会，实现白山新的跨越。

　　到2009年，我市经济社会发展的主要目标是：

　　--努力构建具有白山特点的产业格局，人均主要经济指标在全省领先。调整优化经济结构，拓宽资源开发领域，拉长产业链条，提高资源转换层次和水平，做强能源、林产、矿产冶金产业，培育壮大医药、绿色食品、旅游等产业，创造条件发展技术和资金密集型产业，基本形成具有白山特点的产业格局，国民经济步入快速发展时期。生产总值达到227亿元以上，财政收入达到16亿元以上，年均分别增长12%以上。把白山初步建成国际矿泉城、绿色食品城、优秀旅游城、中国北药生产基地和吉林省乃至东北重要能源基地。

　　--基础设施建设实现新的突破，城镇化水平明显提高。长白山机场、高速公路、地方铁路等重点基础设施建设相继完成，所有乡镇和行政村全部通油（水泥）路，畅通快捷的立体交通网络基本形成，经济地理位置显著改善。中心城市整体功能进一步增强，小城镇带动辐射作用明显提高。

　　--社会文明程度全面提升，各项事业蓬勃发展。科技成果应用广泛，高等教育得到发展，人才兴业战略深入实施，各项社会事业全面进步。全民的文化、道德、法律素质普遍提高，在全社会形成爱国守法、明礼诚信、团结友善、勤俭自强、敬业奉献的良好风气。努力创建全国"双拥"模范城和全国卫生城市。

　　--人民生活质量明显改善，城乡社会保障能力显著提高。城镇居民人均可支配收入和农民人均纯收入年均分别增长10%和7%。累计开发就业岗位20.5万个，城镇登记失业率控制在4.5%以内。基本建立起覆盖城乡、制度完善、实力较强的社会保障体系。

　　--人与自然更加和谐，可持续发展能力进一步增强。着力实施生态保护、恢复和治理工程，人居环境不断改善。推进经济增长方式转变，大力发展循环经济，积极构建节约型社会，可持续发展能力不断增强。人口自然增长率控制在3‰以下。

　　为实现上述目标，我们要牢牢把握以下基本要求。

　　第一，坚持把发展作为执政兴市的第一要务。用科学发展观统领经济和社会发展全局，统领资源型城市发展接续产业示范基地的各项工作，在全市上下形成聚精会神搞建设、一心一意谋发展的浓厚氛围，变"快走"为"快跑"。

　　第二，坚持把提高人民生活质量作为加快发展的根本目的。切实把广大人民群众的利益实现好、维护好、发展好，高度重视、认真解决好涉及人民群众利益的突出问题，在为民办实事上下功夫，让改革和发展的成果惠及千家万户。

　　第三，坚持把改革开放作为加快发展的不竭动力。用开放的思维、改革的办法和创新的精神，充分调动各方面的积极性和创造性，着力解决发展中的关键问题，争取在重点、难点问题上取得突破，增强发展的动力和活力。

　　第四，坚持把维护稳定作为加快发展的重要保障。把维护政治安定和社会稳定作为一项长期的政治任务，妥善处理好改革发展中出现的各种矛盾和问题，为实现大发展营造安定祥和的社会环境。

　　第五，坚持把科学、民主、依法行政作为政府自身建设的根本要求。以建设服务型政府为目标，转变政府职能，改进工作作风，遵循客观规律，充分发扬民主，坚持依法办事，为经济社会发展创造良好的政策、法制和服务环境。

　　三、2005年工作的建议

　　今年是全面实现"十五"计划目标、谋划"十一五"发展的重要一年，是振兴东北老工业基地的关键一年，也是我市新一届政府的开局之年。综合分析各种因素，2005 年国民经济和社会发展的主要预期目标是：全市生产总值增长12%以上，全社会固定资产投资增长45%以上，财政收入与经济增长保持同步，财政支出增长30%以上，社会消费品零售总额增长6%，城镇居民人均可支配收入和农民人均纯收入分别增长10%和6%以上，人口自然增长率控制在3.01‰以下，城镇登记失业率不超过4.2%。实现上述目标，要重点抓好以下九个方面的工作。

　　（一）突出抓好项目建设。

　　市委、市政府把今年确定为"项目促进年"。我们要继续把项目建设作为政府工作的重中之重，认真抓好一批左右全市经济形势的大项目。全年计划安排千万元以上项目238项，总投资282亿元，计划完成投资65亿元。

　　加快在建项目建设进度。全面落实项目法人责任制，进一步加大资金筹措力度，强化项目管理，保证工程质量，加快建设进度。工业项目要完成嘉懋 60 万吨焦化一期工程、靖宇2万吨工业硅生产线、大通矿业30万吨铁精粉、临江健今药业新药产业化等23个项目建设，力争年内投产；松江河发电厂双沟和石龙电站、喜丰节水器材二期扩建、永利葡萄籽综合开发等22个项目完成年度计划。基础设施项目要确保辉南至靖宇地方铁路、靖宇至小西头公路、八道沟至十四道沟公路、老岭隧道及临江至石人段沈长公路建成通车，完成3个乡镇、30个行政村通油（水泥）路工程，白山货运枢纽站投入运营，十四道沟至长白防护工程、县城电网改造和曲家营水利枢纽配套工程年内竣工；南线公路、长白山南坡公路、鸭绿江界河堤防等18个项目完成年度建设计划。

　　促成一批重大项目尽快开工。投资 35 亿元的浑江发电公司60万千瓦机组扩建、投资 5 亿元的大横路钴矿、投资 55 亿元的林浆纸一体化以及奥迪 C6轿车门内板焊接总成、新宇煤矿马当二井扩建、抚松枫林水利枢纽工程等重点项目全面开工。上海华宝投资集团10万吨硅铁一期工程，争取当年建成投产。投资8.3亿元的采煤沉陷区综合治理工程，年内完成工程总量的50%以上。总投资2.6亿元的靖宇国防公路和八道沟至十四道沟防护工程年内完工。总投资7.8亿元的长白山机场和环长白山公路年内破土动工。总投资100亿元的营城子至松江河高速公路和马鹿沟至八号闸边防公路争取尽快开工。

　　积极谋划争取一批后续项目。深入研究国家有保有压的宏观调控政策，围绕省政府重点培育的能源、冶金、建材和森工、纺织、旅游等特色产业，结合我市资源优势和产业基础，精心策划、论证、包装一批带动作用强、辐射范围大、发展后劲足的大项目，真正形成数量充足、结构合理、质量较高、动态开放的项目储备体系。积极促成与香港俊益、绿岛集团、天津顶新集团等合作开发百万吨矿泉水项目。争取将总投资18.3亿元的12 万立方米高密度纤维板等16个项目，列入国家第三批振兴东北老工业基地改造计划。投资23亿元的松江河抽水蓄能电站完成可研。投资 400 亿元的靖宇核电站项目和投资73亿元的千里堤防白山段建设工程年内完成初可研论证。加快靖抚煤田、长白煤田的勘探步伐。靖宇至松江河地方铁路、长白长松岭隧道等项目要加快前期工作进度。

　　(二) 努力提高国民经济运行质量。

　　以提高质量和效益为核心，加快经济增长方式转变，推进国民经济持续健康快速发展。

　　促进工业经济健康发展。以更加优惠的政策和更加完善的服务，支持中省属企业发挥管理和技术装备优势，扩大生产规模，拓展经营领域。加大对喜丰塑料、天风制药、靖宇娃哈哈等 20 户地方重点企业的监控和调度，积极帮助他们解决生产经营中的实际困难，引导企业加强管理，盘活资产，挖掘潜力，提速增效。把扶持中小企业发展摆在重要位置，加强服务、培训和指导，提高企业市场竞争和抵御风险的能力，不断做大做强。组织专门力量，深入调查研究，认真抓好困难企业的扭亏脱困和 "双停"企业的启动工作。全市规模以上工业增加值增长18%，利润增长15%。

　　大力发展农业和农村经济。高度重视"三农"工作，全面提高农业效益，进一步增加农民收入。加大农业和农村经济结构调整力度，加快发展人参、山珍绿色食品和畜牧业等特色产业，努力开发培育名牌产品，不断提高知名度和市场占有率。积极推动主要农产品优质化、专用化进程，力争高效作物占有率和种植业优质品率分别达到50%。重点培育西洋参集团、万隆猪场、大阳岔食用菌公司等龙头企业，提高农业产业化水平。加快发展各类农村经济合作组织和经纪人队伍，提高农民的市场化组织程度。继续加大对农村基础设施的投入，增强抵御自然灾害的能力。兴建30处农村安全饮水工程，解决部分乡镇和村屯人畜饮水困难。加强林政管理，严厉打击各类破坏森林资源的违法犯罪行为。进一步完善森林火灾预防和扑救体系，确保实现连续 26 年无重大森林火灾。

　　发展壮大以旅游业为牵导的第三产业。按照《白山市旅游发展总体规划》，加快景区、景点和松江河综合服务区等旅游配套设施建设。继续办好长白山踏青旅游节、野生花卉节、金秋红叶节和冰雪旅游节，积极开发民俗旅游和红色旅游，提高接待能力和整体效益，旅游社会总收入增长39.4%。继续巩固提升商贸流通、餐饮服务、交通运输等传统行业，积极发展现代流通、信息、通信、中介等新兴行业，第三产业增加值增长13.6%。

　　做好财税金融工作。强化税收征管，打击偷逃税行为，确保财政收入稳定增长。继续调整和优化支出结构，加强预算外资金管理。进一步强化审计监督。努力提高企业资产质量和信用等级，增强金融对地方经济发展的支持力度。抓紧组建地方商业银行。完成市中小企业信用担保公司增资扩股，提高担保能力。支持农村信用合作社发展，建立健全农村小额信贷机制。加强金融监管，防范金融风险。

　　全力实施县域突破。按照市域有规模、县域有特点、城乡一体、统筹发展的思路，加强对县域突破的组织领导，推动各县（市）区发挥比较优势，培育特色产业，增强县域经济实力。建立以中心城市为核心、县城为基础、小城镇为依托，层次分明、功能互补的城镇发展体系，尽快形成以工促农、以城带乡的发展格局。

　　（三）继续深化各项改革。

　　认真落实全省国有企业改革工作会议精神，按照有利于企业发展、有利于维护职工利益、有利于调动改革者积极性的要求，采取产权出售、招商重组、引资改造、债权转股等多种形式，全面推进企业改制，做到整体改制到位、债权债务处理到位、职工劳动关系转换到位、国有资本退出到位，实现体制和机制创新。充分用好用足国家和省有关政策，通过国有股权转让、资产变现、土地置换、减免有关收费等方式，千方百计筹集改革成本，确保改革顺利推进。年底前，尚未改制的92户市、县属国有和集体企业全部完成改制任务。抓住商业银行进行股份制改造和四大资产管理公司集中处置不良资产的机遇，通过政策性破产、依法破产、诉讼清偿等核呆形式，基本完成全市企业核销呆帐贷款。按照现代企业制度的要求，完善公司法人治理结构，建立决策追究和对企业经营者业绩考核评价制度，促进股份制企业依法运行。继续深化农村税费改革，把免征农业税、取消特产税和农民义务工政策落实到位。积极稳妥地推进粮食流通体制改革。切实抓好林地使用权、林木所有权的确权发证工作，实施林业分类经营管理体制改革。加快农村教育综合改革和农村义务教育管理体制改革步伐。深化医疗卫生体制改革。认真抓好政府机构改革和事业单位人事制度改革。

　　（四）全方位扩大对外开放。

　　牢固树立招商引资的争先意识，围绕主导产业和希望产业发展，搞好招商引资规划，推出一批优势资源开发、基础设施建设等方面的招商项目，以高质量的项目，提高招商引资成功率，增强我市利用内资外资的吸引力。坚持走出去与请进来相结合，在巩固赴北京、深圳和韩国招商引资成果的基础上，积极参加省组织的国内外大型经贸活动，精心组织好赴"长三角"、韩国和日本的招商活动。以矿泉节和旅游节为有效载体，有重点、有选择地邀请客商，到白山实地考察、洽谈项目、投资兴业。坚持大招商、招大商，以引进国内外大公司、大集团等战略投资为重点，广泛寻求合作伙伴，实现互利双赢，共同发展。建立招商引资工作目标责任体系，层层分解落实责任目标，强化跟踪调度和督查考核，加快健全在政府推动下，以企业为主体、以项目为载体、市场化运作的招商引资新机制，形成与对外开放相适应的全方位、宽领域、多元化的招商引资新格局，开创我市招商引资工作新局面。全年实际到位资金15亿元。加强开发区建设与管理，形成各具特色的产业体系，使之成为引资引智的重要平台。增加出口产品种类，大力开拓国际市场，搞好边境互市贸易，确保外贸进出口总额突破1亿美元。

　　（五）大力发展民营经济。

　　牢固树立亲商、富商、安商、扶商观念，努力在全市营造尊重财富、崇尚创业的致富氛围。认真贯彻《国务院关于鼓励、支持和引导非公有制经济发展的若干意见》。通过放宽市场准入条件、简化和规范审批登记手续、实行税收优惠政策、拓宽民营企业融资渠道、鼓励民营企业参与国企改革、建立健全服务体系等措施，加强扶持和引导，推动民营经济做大做强。以提高民营经济的产业层次和企业素质为核心，改进服务和加强监管，积极鼓励、支持和引导民营经济发展。继续实施"扶优促强"工程，鼓励规模大、辐射带动能力强的骨干民营企业，参与国有和集体企业的改制、改组、改造，参与国有经济战略性调整，使民营经济发展既得到总量的扩充，又向质的提高转变。立足本地的产业特色，积极引导域外民营企业到白山投资兴业，大力培育一批当地的民营企业"老板"，发展壮大民营企业群体，不断提升民营经济在国民经济中的比重。引导民营企业在提高经济效益的基础上，增加积累和科技投入，加快科技进步，尽快改变装备比较差、工艺比较落后、产品档次比较低的现状。积极搭建融资平台，帮助民营企业解决创业初期和经营过程中资金不足问题。适时组织民营企业家到发达地区考察、学习、交流，更新经营理念，增强创新意识。规范政府执法行为，维护民营企业的合法权益。

　　（六）不断完善城市功能。

　　坚持高起点规划、高标准建设、高效能管理，全面提升城市形象。

　　提高城市规划水平。按照城市总体规划要求，完成城市交通规划、环卫设施发展规划、园林绿地系统规划编制工作，完善江北新区城市设计，编制北线公路以北环境总体整治规划和南山区修建规划，市区控制性详细规划覆盖率力争达到80%。

　　加快城市基础设施建设。严格执行建设用地集中供应和土地使用权公开交易制度，大力推进城市设施多元化投入、市场化经营，拓宽融资渠道，配套完善城市基础设施，增强综合服务功能。加快江北新区开发步伐，完成江畔明珠小区15万平方米住宅建设，市区新开发房地产面积30万平方米以上。完成星泰大桥、向阳路过江悬索桥和钢板闸收尾工程，改造浑江北岸江堤并建成彩虹桥上游橡胶坝，完成市垃圾处理场一期工程，开工建设市区集中供热三期工程，改造市区部分供水管网，做好市污水处理厂开工前准备工作。建成卫国路、南平街、喜丰路和通沟街与南线公路接口，开工建设靖宇路、东盛路和河口街与南线公路接口，完善市区路网结构。坚持"既要锦上添花，更要雪中送炭"，继续改善基本群众的生产生活环境，加大江南旧城改造力度，突出抓好平房和旧房集聚区、背街小巷和城乡结合部基础设施建设，市区新建街路巷道14条、维修改造6条，铺设方砖7.7万平方米，安装路灯268盏，新建、改建公厕106处。改造北山公园，以长白山特有植物为主进行配置。抓好江畔明珠小区绿化带、滨江公园西段建设和浑江大街、滨江西街草坪改造，更换浑江南岸江堤通江桥至金坑河段方砖。加快县城、中心镇和旅游沿线小城镇建设。

　　全面加强城市管理。进一步理顺城市管理体制，完善各项制度，逐步建立城市管理长效机制。发动社会各界力量，采取集中整治与常态管理相结合的方式，继续开展市容市貌综合整治活动，抓好环境卫生、公共秩序和物业管理。强化市民的养成教育和参与意识，形成齐抓共管的局面，全面提高管理水平，达到省级卫生城市标准。

　　（七）全面推进精神文明建设和社会各项事业发展。

　　强化文明养成教育，努力提高公民素质，繁荣各项社会事业。

　　加强精神文明建设。认真贯彻落实《公民道德建设实施纲要》，深入开展形式多样的精神文明创建活动，继续抓好城市"六进社区"、农村"一推双带"、"双拥"和军（警）民共建活动，全面提高市民素质和城乡文明程度。依法加强文化市场管理，加大"扫黄"、"打非"力度，认真组织好网吧等互联网上网营业场所专项治理。加强长白山文化研究与开发，深入开展社区文化、农村大文化和广场文化建设，活跃群众文化生活。进一步健全学校、社会、家庭三位一体的教育网络，突出抓好理想信念教育，加强和改进未成年人思想道德建设。

　　全面发展社会各项事业。坚持科技创新与经济发展紧密结合，围绕我市重点发展的产业，搞好产、学、研对接，努力提高科技成果转化率。进一步整合教育资源，优化教育结构，大力推进教育手段现代化，全面提升教育质量。认真落实区域卫生规划，不断完善公共卫生体系。建成市中心医院住院大楼，改善市区就医环境。加强乡镇医疗卫生基础设施建设，提高诊疗水平。继续加大对广播电视事业的投入，完成市级有线数字电视平台建设和网络改造，开播数字电视节目。优化人口出生质量，稳定低生育水平。认真做好省第十五届运动会和全国第十一届冬运会的参赛准备工作。广泛深入开展群众性体育活动，完成市全民健身中心建设，改善群众健身条件。加快民族地区发展步伐。依法加强对宗教事务的管理。切实维护妇女儿童的合法权益。加强对矿山开采、公路建设等造成的地质和植被破坏的恢复。进一步强化对污染源的综合治理，不断改善人民群众的生活环境。建立抚松天然矿泉水水源保护区，编制完成长白天然矿泉水水源保护区规划。加强防震减灾体系建设，提高防震减灾能力。

　　（八）切实维护人民群众根本利益。

　　高度关注民生，千方百计扩大就业，进一步加大社会保障和扶贫解困力度，认真做好信访、安全生产和社会治安工作，为人民群众创造安居乐业的社会环境。

　　认真抓好就业再就业工作。坚持用加快发展的办法解决就业再就业问题，按照劳动者自主择业、市场调节就业和政府促进就业的方针，认真落实各项优惠政策和全民创业促就业各项措施，健全公共就业服务制度，认真做好就业培训和指导工作，鼓励、引导下岗失业人员自谋职业和自主创业。广开就业门路，大力发展民营经济、社区服务业、中小型企业和劳动密集型企业，进一步扩大劳务输出规模。依法规范企业裁员行为，保障劳动者的合法权益。全年新开发就业岗位3万个，城市新增就业2.55万人，下岗失业人员再就业1.76万人。

　　不断增强社会保障能力。积极稳妥地推进完善城镇社会保障体系试点工作，加大政策宣传力度，指导、帮助企业落实并轨条件，按计划完成试点工作任务。加强社会保障体系建设，进一步扩大养老、失业、医疗、工伤保险覆盖面，加大基金征缴和清欠力度，提高支付能力。抓好住房公积金归集工作，为广大职工改善住房条件提供资金保障。做好启动生育保险的基础性工作。加强城乡社会保障救助体系建设，努力提高城乡居民最低生活保障水平。

　　营造安定祥和的社会环境。继续深化同"法轮功"等邪教组织的斗争，严厉打击境内外敌对势力的渗透和破坏活动，确保政治安定。开展有针对性的专项斗争，重点打击带有黑社会性质的犯罪和杀人、爆炸、抢劫、抢夺、盗窃犯罪，集中打击赌博违法犯罪和非法传销活动。强化社会治安综合治理，完善防控体系，减少可防性犯罪的发生。加强边境建设和管理。继续抓好以煤矿为重点的安全生产工作，切实加强非煤矿山、交通运输、危险化学品、消防、校园和食品卫生等安全管理，坚决防止各类重特大事故的发生。全面落实信访工作责任制，建立联合接访、共同参与的大信访工作格局，变上访为下访，超前排查、化解各类矛盾，认真负责地解决群众反映的突出问题。

　　（九）进一步提高政府行政能力和水平。

　　各级政府要履行好经济调节、市场监管、社会管理和公共服务职能，提高加快发展、协调发展、稳定发展的能力，推动全市经济社会持续快速健康发展。

　　提高素质、转变作风。认真开展保持共产党员先进性教育活动，强化公务员培训，鼓励在职学习，支持进修深造，选派挂职锻炼，教育广大干部坚持科学的发展观，树立正确的政绩观，努力建设高素质的公务员队伍。进一步改进政风，倡导开短会、讲短话、发短文，加快工作节奏，提高行政效能，集中精力抓大事、抓难事、抓落实。各级干部要走出机关、重心下沉，积极主动地为基层、企业和群众解决实际问题，重实干、办实事、求实效。要关心、支持和重用那些敢讲真话、能干实事、勇挑重担、善解难题的好干部，对那些思路不清、办法不多、措施不力、效率不高、在其位不谋其政的干部，要建议市委采取组织手段予以调整。

　　推进科学、民主、依法行政。坚持从实际出发，按客观规律办事，逐步建立和完善公众参与、专家论证和政府决策相结合的决策机制，努力推进行政决策的科学化、民主化和制度化。既要协调处理好当前的突出问题和矛盾，又要注意解决好长期性、战略性问题，认真做好全国第一次经济普查工作，高标准完成"十一五" 规划编制工作，谋划好今后五年的发展。围绕为民、利民、便民，全面实行"阳光政务"，突出解决集中审批、联合办理、规范服务和信息公开、加强监督等问题。办好市长和县（市）区长公开电话，畅通政府与群众联系的渠道。进一步提高市政务服务中心管理水平，加快县（市）区政务服务中心建设，对所有符合条件的行政审批和行政事业性收费，都要纳入各级政务服务中心集中办理，规范运行。进一步落实政府新闻发布和重大决策、重要事项的公开咨询、公开听证和公示等制度，建成市政府门户网站、重点部门和县（市）区政府网站，增强政府工作透明度，提高政府公信力。加强政府法制建设，自觉接受人大、政协的法律监督、工作监督和民主监督。加快配套制度建设，理顺行政执法体制，规范约束行政权力，提高政府组成人员和工作部门依法行政、公正执法的能力，着力解决职能交叉、审批程序复杂、多头执法、重复处罚、执法扰民等问题，真正把政府经济管理职能转到主要为市场主体服务上来。

　　优化经济发展软环境。各级政府要根据新的情况，继续清理规范性文件和收费项目，研究制定更多的鼓励支持全民创业、招商引资、人才流动等政策，尊重、善待、支持投资者和创业者，为他们排忧解难，提供优质服务，努力在全市上下形成千方百计支持发展、推动发展、加快发展的浓厚氛围。着力建设"诚信政府"，制定的政策要落实到位，做出的承诺要严格兑现。建立企业综合信用评价体系，实行信用信息公示制，构筑诚信社会，打造"信用白山"。整顿和规范市场经济秩序。加大对涉软案件的查处力度，谁破坏软环境就砸谁的饭碗。

　　继续加强廉政建设。坚持标本兼治、综合治理，惩防并举、注重预防的方针，建立健全教育、制度、监督并重的惩治和预防腐败体系。深入开展示范教育和警示教育，继续抓好领导干部廉洁自律，进一步加强对各级领导班子特别是"一把手"的监督，尤其要加强对手中掌握一定权力的领导干部和工作人员的监督。深入开展民主评议政（行）风和软环境"双评"活动，坚决纠正部门和行业不正之风。严肃查办大案要案，继续保持对腐败分子的高压态势，树立和维护政府的良好形象。

　　各位代表！新的形势催人奋进，新的事业需要我们奋力开拓。让我们在省委、省政府和市委的正确领导下，凝聚全市各族人民的智慧和力量，励精图治，开拓创新，真抓实干，把白山经济和社会各项事业推向一个新的发展阶段。
